# Testing the Accuracy of Aerial Surveys for Large Mammals: An Experiment with African Savanna Elephants (*Loxodonta africana*)

**DOI:** 10.1371/journal.pone.0164904

**Published:** 2016-10-18

**Authors:** Scott Schlossberg, Michael J. Chase, Curtice R. Griffin

**Affiliations:** 1 Elephants Without Borders, PO Box 682, Kasane, Botswana; 2 Department of Environmental Conservation, University of Massachusetts Amherst, Amherst, Massachusetts, United States of America; University of Illinois at Urbana-Champaign, UNITED STATES

## Abstract

Accurate counts of animals are critical for prioritizing conservation efforts. Past research, however, suggests that observers on aerial surveys may fail to detect all individuals of the target species present in the survey area. Such errors could bias population estimates low and confound trend estimation. We used two approaches to assess the accuracy of aerial surveys for African savanna elephants (*Loxodonta africana*) in northern Botswana. First, we used double-observer sampling, in which two observers make observations on the same herds, to estimate detectability of elephants and determine what variables affect it. Second, we compared total counts, a complete survey of the entire study area, against sample counts, in which only a portion of the study area is sampled. Total counts are often considered a complete census, so comparing total counts against sample counts can help to determine if sample counts are underestimating elephant numbers. We estimated that observers detected only 76% ± SE of 2% of elephant herds and 87 ± 1% of individual elephants present in survey strips. Detectability increased strongly with elephant herd size. Out of the four observers used in total, one observer had a lower detection probability than the other three, and detectability was higher in the rear row of seats than the front. The habitat immediately adjacent to animals also affected detectability, with detection more likely in more open habitats. Total counts were not statistically distinguishable from sample counts. Because, however, the double-observer samples revealed that observers missed 13% of elephants, we conclude that total counts may be undercounting elephants as well. These results suggest that elephant population estimates from both sample and total counts are biased low. Because factors such as observer and habitat affected detectability of elephants, comparisons of elephant populations across time or space may be confounded. We encourage survey teams to incorporate detectability analysis in all aerial surveys for mammals.

## Introduction

Worldwide, large mammals are under threat due to habitat loss and fragmentation, overharvest, and human-wildlife conflict [1–4]. Because resources for conservation are limited, accurate population estimates are needed to determine trends in mammal populations and to guide interventions for maximum benefits [5]. For declining species, inaccuracy or bias in population estimates is not just an academic issue but can actually hinder conservation by causing misallocation of scarce resources [6]. In time series of population estimates, poor survey data can introduce spurious trends or obscure real ones [7–9]. Thus, determining the accuracy of survey methods for mammals is critical.

Because of their large geographic ranges, in open habitats, large mammals are typically counted via aerial survey (e.g. [10]). An important but rarely acknowledged assumption of standard aerial surveys is that all animals in the survey area are detected [5,11]. Past research, however, suggests that aerial surveys may underestimate numbers. Caughley [11] reported that just 61% of large mammals were detected on aerial surveys, and subsequent studies have found similar results [12–14]. Observers can miss animals on aerial surveys for a variety of reasons including lack of skill or training, fatigue, inattention, dense vegetation, the demands of counting multiple species, interference from the sun, and excessive speed or altitude in the survey aircraft [15–18].

Aerial surveys for large mammals usually follow one of two sampling schemes: total counts or sample counts [19]. Total counts involve counting all animals in the survey area by flying along closely spaced transects. Sample counts survey a subset of the study area, usually 5–20%, by flying widely spaced transects with narrow survey strips and then extrapolating to the larger survey area. Though sample counts are asymptotically unbiased, the population estimate from any individual count will deviate from the true value due to sampling error [20]. Thus, total counts are often considered more precise and accurate than sample counts [21,22]. Nonetheless, almost no studies have tested the assumption that total counts are more accurate. Total counts in open habitats typically have transect spacing of 1 km [23], which implies that observers must search 500 m on either side of the plane. Experiments have shown that the number of animal detections per unit area surveyed decreases with strip width [11,15]. Given the wide usage of total counts in aerial surveys, the possibility that total counts are undercounting animals needs to be tested.

Today, survey protocols for many animals and even some plants incorporate corrections for detectability (e.g. [24–26]), but for large mammals, detectability is often ignored. To improve the reliability of data on mammal populations and aid in conservation efforts, we tested the accuracy of aerial surveys for large mammals using African savanna elephants (*Loxodonta africana*) as a study species. We used double-observer aerial surveys to estimate detectability of elephant herds and determine what variables affect it. We also compared total counts with sample counts for the same survey areas to determine if total counts are more accurate. Our goals were to determine the detectability of elephants and effects of covariates such as observer, habitat, and flight speed on detectability. We also sought to provide suggestions for improving future elephant surveys, and to learn whether or not elephant population estimates show any systematic bias.

## Materials and Methods

### Study area

**Fig 1:**
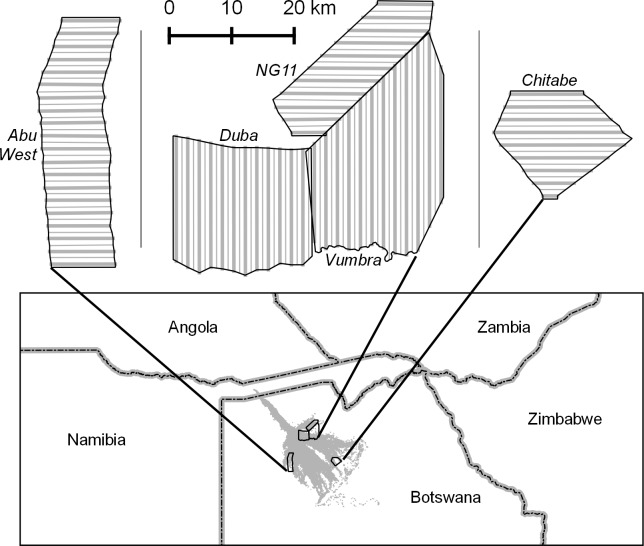
Study areas for elephant surveys in Botswana. Stratum names are shown in italics. Thick gray lines denote transects spaced 2 km apart used in sample counts with double-observer sampling. Transects used in helicopter total counts were 1 km apart and followed both thick gray lines and thin gray lines. Shaded area in lower figure indicates the Okavango Delta.

We conducted aerial surveys for elephants in the Okavango Delta of northern Botswana. Our study area consisted of five study areas or strata ranging from 236 to 545 km^2^ in size (Fig 1). Each stratum encompassed part or all of a concession used for wildlife viewing. On these sites, elephants occur in a variety of habitats including marsh, grassland, savanna, woodland, and shrubland. Human impact on these sites was minimal, though there were jeep tracks and safari camps in some strata. Permission to fly surveys was granted by the Botswana Department of Wildlife and National Parks.

### Double-observer surveys

To estimate detectability, we used double-observer surveys in which two observers on the same side of the aircraft independently and simultaneously counted elephants in the same survey strip [27]. There were three possible outcomes for each herd observed: a) seen by the front observer only, b) seen by the rear observer only, or c) seen by both observers. By analyzing the frequency of these outcomes, one can determine the probability of detecting a herd and test covariate effects on detectability (see below).

We flew double-observer flights in a GippsAero GA8 Airvan, which was ideal for our purposes because of its large windows and four rows of two seats. During surveys, we had two sets of paired observers, one pair on each side of the aircraft. The pilot and the front recorder sat in the front two seats. In the second row were two “front” observers, one on either side of the plane, and in the third row were two “rear” observers. The rear recorder sat in the fourth row. Note that we refer to observers as “front” and “rear” throughout based on their relative positions; the observers were actually in the second and third rows of seats. One assumption of our analysis is that front and rear observers make observations independently, without cuing on the other observer. To visually isolate front and rear observers, we hung an opaque cloth from the cabin ceiling behind each front observer. Each crew member wore headphones connected to an intercom system. The rear recorder and observers had an isolated intercom system so that front and rear observers could not hear one another speak. The four observers rotated positions between days so that each observer conducted at least one day of surveys in each seat.

Double-observer flights were flown using the sample transect method, a standard sampling scheme for aerial surveys [19]. In each of the five strata, we established a set of parallel transects spaced 2 km apart (Fig 1). The pilot flew along these transects sequentially, with the direction of travel alternating between adjacent transects, and observers recorded animals in strips on either side of the plane. Metal wands attached to the wing struts demarcated the strips, which were calibrated on the ground to be 200 m wide at 91.4 m altitude. Because realized strip width can vary with the observer’s head position, we placed tape on each window to ensure consistent eye position. We flew a series of calibration flights to determine actual strip widths, following the procedures of Frederick et al. [23].

Double-observer surveys took place from 28 July to 1 August 2014, during Botswana’s dry season. Deciduous plants were leafless at this time, allowing good viewing conditions. Also, elephant movements are relatively restricted at this time [28]. We surveyed 1–2 strata per day. The stratum surveyed on day 3 was surveyed again on day 5, when observers recorded only elephant herds (see below). Surveys took place between 0900 and 1300 hrs. The pilot was instructed to fly at 91 m above ground and 180 km/hr. We used a custom data logger to record groundspeed as measured by a GPS receiver and altitude as measured by a laser altimeter. Transects averaged 16.4 km in length (range: 2.4–31.3 km) and required a mean of 5.8 minutes to survey.

The pilot navigated by GPS, and the front recorder monitored the GPS to determine when transect start/end points were reached. The front recorder used hand gestures to signal the rear recorder to tell the rear observers to start or stop counting. While on transects, observers called out sightings by species and herd size, and the recorders obtained a GPS fix for each herd and recorded the sighting on a data sheet. We used digital audio recorders connected to the intercom systems to verify the written observations. The plane was equipped with four high-resolution digital SLR cameras, one for each observer, mounted in the windows and focused on the survey strips. Observers were instructed to use a remote shutter button to photograph each herd sighted. We used photographs to reconcile observations between front and rear observers and to correct herd-size estimates.

During the first four days of surveys, observers were instructed to count all medium and large mammal species in the survey strips. We included species besides elephants because most aerial surveys in Africa are multi-species surveys. To determine if searching for multiple species affected elephant detections, on the fifth and final day of surveys, observers were instructed to search for only elephants and no other species.

The four observers varied substantially in previous experience with aerial surveys. Observers 1 and 3 had ~1000 hrs of experience conducting aerial surveys in Africa. Observer 2 had ~150 hrs of previous experience. Observer 4 was highly experienced in identifying large mammals in southern Africa but had no previous experience with aerial surveys. Before data collection began, observer 4 received 5 hrs of airborne training in conducting surveys to increase his familiarity with the procedures.

### Total counts

We conducted total counts on the same five study sites used in the double-observer study. Total counts were flown in a Robinson Raven helicopter with a crew of four: pilot, recorder, and two rear-seat observers (1 and 2 from the double-observer study). We flew surveys with the rear doors removed to facilitate viewing. As above, observers called out sightings of all medium and large mammals seen to the recorder who transcribed the data. The recorder also made sightings opportunistically. We conducted total counts the week prior to the double-observer counts, between 21 and 27 July 2014.

To ensure complete coverage of the study area, the helicopter flew along transects spaced 1 km apart rather than the 2 km used in the double-observer study. Researchers recommend spacing of 1 km for total counts in open African habitats [23]. The total counts used the same transects as the double-observer counts as well as an additional transect between each pair of double-observer transects (Fig 1). No survey strip was delineated on total counts; observers counted all wildlife in view. The helicopter deviated from the transects when observers needed to obtain better views of herds. Each observer had a camera to photograph larger herds for photo correction. Because, however, the helicopter could hover and circle as necessary to count animals, photographs were unnecessary for most herds.

### Double-observer analysis

Our unit of analysis for the double-observer analysis was the herd, as research has shown that herd-forming animals tend to be detected as a group rather than individually [29]. Most elephant herds observed were well-defined and clearly separated from one another. Where we were uncertain about herd boundaries, we arbitrarily divided groups into separate herds where they were separated from one another by the width of one photograph (approximately 500 m), with no intervening elephants. Using photographs (available for 91% of herds observed), we matched herd observations between front and rear observers to determine if a herd had been seen by one or both observers. For observations without photographs, we used the sexes (bull vs. breeding herds), herd sizes, and times of the GPS fixes for the front and rear observers to align sightings.

We also used the photographs to correct observers’ estimates of herd size. First, we corrected each observer’s herd size estimates independently, ignoring any information from the other observer on the same side of the plane. We only reduced herd size estimates below the original estimate if a photograph clearly showed the entire herd. If individual animals were potentially left out of the photograph or obscured by vegetation, we did not lower a herd-size estimate. For small herds, where the original herd size estimate was ≤6, we assumed that observers counted accurately and did not adjust herd counts downward [30]. Otherwise, we adjusted herd counts based on the number of individuals visible in the photographs.

After we independently corrected herd sizes for each observer, we reconciled the herd size estimates for herds seen by both observers. If no photos were available for either observer, we simply took the mean of the two estimates, rounding up for fractions. If photos were only available for only one observer, we used the photo-corrected estimate. If photos were available for both observers, we attempted to combine the two photographs to determine the total number of individuals present. Because photographs from each observer were usually taken at slightly different times, the distinct viewing angles allowed us to determine which individuals were missed by each observer for some herds. After accounting for each individual, we used the count of all individuals based on both photographs as the herd size.

We modeled detectability with the closed-population recapture model developed by Huggins [31,32]. This model uses maximum likelihood to estimate the probability of detection by conditioning on the unknown number of herds missed by both observers. The Huggins model allows detectability to be parameterized as a logistic function with continuous or categorical covariates for individual herds. The basic equation for the detectability function was $p_{i}=(1+e^{-X_{i}\beta }{)}^{-1}$, where *p*_*i*_ is the detection probability for herd *i*, **X**_***i***_ is a row vector of covariate information for herd *i*, and **β** is a column vector of coefficients to be estimated. We implemented models using Program MARK [33].

Based on previous research, we identified several variables that could affect detectability of elephants (Table 1). We used a two-stage information-theoretic process to determine which variables were supported by the data [34,35]. In the first stage, we pre-screened all variables against a constant-only model. Most of the pre-screened variables could be modeled with a single parameter plus an intercept, but some, such as the model with distinct detection probabilities for each observer, had multiple parameters. Variables that were pre-screened fell into seven categories (Table 1). For position in the plane, we tested 1) front versus rear row because visibility may differ by row, 2) an effect of the rear-left seat because that window was slightly smaller than the other observers’ windows, and 3) all four seats individually [17]. Models for observer effects included a model with unique parameters for each of the four observers and a model for each observer in which that observer had a different detection probability than the other three. We used this formulation because we expected observer 4, with no previous experience, to have less ability to detect elephants than the other three observers [16]. We modeled four different types of fatigue: within transects (time since start of transect), within days (time since beginning of day’s surveys), across days (number of days since day 1), and an interaction between the within-transect and within-day effects, testing if within-transect fatigue increased as the day progressed [18]. Because the final day’s sample included only elephants, we tested effects of counting elephants versus all species. Past research has shown that flight parameters can affect detectability, so we tested groundspeed, altitude, and an interaction between the two [15]. Because larger groups have been more detectable in past studies, we tested a linear effect of herd size [17]. Finally, we tested whether or not the azimuth of the sun and its height above the horizon affected detectability [18].

**Table 1:** Covariates screened for effects on detectability in double-observer aerial surveys of elephants in northern Botswana.

Category	Model	Description	K
Observer	All 4 observers	Separate parameter for each observer	4
	Obs. 1	Observer 1 vs. observers 2, 3, and 4	2
	Obs. 2	Observer 2 vs. observers 1, 3, and 4	2
	Obs. 3	Observer 3 vs. observers 1, 2, and 4	2
	Obs. 4	Observer 4 vs. observers 1, 2, and 3	2
Position in plane	Side	Left vs. right side	2
	Rear-left	Rear-left seat vs. all others	2
	Row	Front vs. rear rows	2
	Each position distinct	Separate parameter for each position	4
Fatigue	Across days	Linear effect of day number	2
	Within day	Linear effect of time since start of day	2
	Within transect	Linear effect of time since start of transect	2
	(Within day) * (within transect)	Interaction between time of day and time since start of transect	2
Herd size	Herd size	Linear effect of herd size	2
All species vs. elephants only	Elephants only	Parameter for day when only elephants were counted vs. days counting all species	2
Flight parameters	Speed	Linear effect of ground speed	2
	Height	Linear effect of height above ground	2
	Speed * height	Speed by height interaction	2
	Direction	Aircraft heading: north, south, east, or west	4
Sun	Sun elevation	Linear effect (degrees)	2
	Relative azimuth	Sun azimuth relative to observer	2
	Elevation * relative azimuth	Interaction between azimuth and elevation	2

K, number of parameters. All models were linear on a logistic scale.

We tested each variable in Table 1 against the constant-only model by comparing values of Akaike’s information criterion corrected for small sample size (AIC_c_) [36]. Variables with a lower AIC_c_ than the constant-only model passed the initial screening. In the second stage, we generated models with all possible additive combinations of variables that passed the first stage. We excluded models with redundant parameters, such as a model with parameters for front vs. rear position and parameters for each of the four seats. Our primary goal was to determine which variables affected elephant detectability, not to choose a single best model. Thus, we ranked the final models by AIC_c_ and then used model-averaging on the top 90% of models by weight to make inferences about variables. We considered variables supported if they had weight of evidence (sum of Akaike weights) >0.5 and strongly supported if they also had model-averaged parameters with 85% confidence intervals (CI) that did not include 0 [37].

We next attempted to estimate the proportions of herds and individuals present in the sample strips that were detected by each observer (not to be confused with the probability of detecting a single herd, which we refer to as “detectability” or “detection probability”). This required us to estimate the number of herds and individuals missed by observers. We used the model-averaged parameter estimates to calculate detectability for each observer and herd size. We then estimated the total number of herds in the strip for each observer and herd size as *m*_*i*_ = *n*_*i*_/*p*_*i*_, where *n*_*i*_ is the number of herds of size *i* observed, *p*_*i*_ is the detectability for herd size *i*, and *m*_*i*_ is the corrected number of herds present [31]. The estimated proportion of herds detected by an observer was ∑_*i*_ *n*_*i*_/∑_*i*_ *m*_*i*_. We used the delta method to estimate the SE of this quantity. We used similar calculations to determine the overall proportion of individual elephants present detected by each observer. Multiplying the counts of herds, *m* and *n*, by *i* transforms them to counts of individuals. Thus, the proportion of individuals detected was ∑_*i*_ *n*_*i*_*i*/∑_*i*_ *m*_*i*_*i*. Again, we use the delta method to estimate the SE of the proportion of individuals detected.

To determine effects of habitat on detectability, we developed a simple scheme to classify habitat around elephant herds (Table 2). These categories were meant to be heuristic rather than a detailed description of habitats. Vegetation in the Okavango Delta is highly heterogeneous and patchy on multiple scales. Thus, we chose to focus on only the habitat type immediately around each elephant, defined as a radius of one body length from each animal. For herds where individual elephants were found in different habitat types, we used the modal type for the herd. In cases of ties, we used the habitat that we considered more open *a priori* (closer to the top of Table 2).

**Table 2:** Categories used to classify habitat around elephants and number of herds observed in each category on double-observer aerial surveys.

Category	Description	Number of herds
water	open water	4
bare ground	no vegetation	4
low grass	leaving elephant legs at least partially exposed	51
open shrub	woody plants up to height of adult; canopy cover <50%	117
open tree	woody plants taller than adult; canopy cover <50%	55
tall grass	completely covering the legs or taller	12
closed shrub	woody plants up to height of adult; canopy cover >50%	27
closed tree	woody plants taller than adult; canopy cover >50%	8

Because some habitat categories had too few observations for analysis, we eliminated or merged categories with <10 observations. We combined “closed tree” with “closed shrub,” as both had >50% woody cover. We also combined “bare ground” with “low grass,” as both had no cover obscuring the view of the elephant’s body. We excluded “water” from the analysis because there was no analogous category with which to combine it.

Some elephant herds were not photographed, and the Huggins model does not allow for missing data, so we could not analyze habitat effects along with other covariates. Instead, we applied the results from the above analysis of elephant detectability done with the full dataset. We used AIC_c_ to compare 1) a “null” model including only the supported variables from the two-stage analysis described above and 2) a model including those variables and the five modified habitat categories. We treated “low grass/bare ground” as the reference category in the analysis.

### Total and sample count analysis

As an additional way of determining the accuracy of elephant surveys, we compared population estimates from the helicopter total counts with those form the double-observer sample counts. To analyze the total count data, we first corrected herd-size estimates with photographs. Because the helicopter was able to circle herds, photos were taken for only 22% of the 659 herds observed. For those herds, we followed the same rules in photographic correction as we did for the initial corrections in the double-observer study. To estimate population sizes for total counts, we simply summed the corrected herd sizes for each stratum. Total counts do not have an associated variance.

To compare total counts with sample counts, we treated the double-observer flights as sample counts. Typical sample counts have just one observer on each side of the plane. Because, however, we had two observers on each side of the airplane, we were able to compute three different sample estimates for each stratum: one for front observers’ sightings, one for rear observers’ sightings, and one for combined sightings of front and rear observers. This was advantageous because detection probabilities differed between the front and rear rows, so we could compare population estimates for each row with the total count. We computed population estimates for both rows of seats combined by including all herds sighted by at least one observer. We did this because combining the front and rear observations leads to a sample-count estimate that comes close to controlling for detectability. The detection probability for front and rear observers combined is 1 ‒ (1 ‒ *p*_*front*_)(1 ‒ *p*_*rear*_), where *p* is the detection probability for a position. If *p*_*front*_ and *p*_*rear*_ are >0.8, the combined probability should be >0.94, which allowed us to compare the total count against a sample count with high detectability.

For front, rear, and all observers, we used Jolly’s [38] estimator for unequal transects to estimate population sizes and their variances for each stratum. Accordingly, elephant density in a stratum is the total number of elephants counted divided by the area sampled. To compute the area sampled, we used position-specific strip widths corrected for height above ground level by transect [19]. For the stratum surveyed on day 3 and day 5 (Vumbra; see Fig 1), we computed separate population estimates for each day. For each stratum, we compared the sample estimates with the total count for that stratum with one-sample *z*-tests.

## Results

### Double-observer sampling

Over five days of double-observer sampling, we recorded 308 elephant herds, averaging 5.5 animals per herd (range: 1–69). Individual observers recorded between 119 and 135 herds each.

Of the 22 variables that we initially screened for effects on detectability (Table 1), we retained 5 for further testing: front vs. rear row, seat position (4 separate estimates), rear-left seat vs. other seats, observer 2 vs. the other three observers, and herd size (S1 Table). We tested those five variables in all possible additive combinations to create a final set of 16 models. There was considerable model-selection uncertainty in the final model set, as the top model had weight = 0.50 (S2 Table). Model averaging indicated strong support for effects of herd size, front vs. rear seat, and observer 2 on detectability (Table 3). Observer 2 had the third most experience of the observers in our study.

**Table 3:** Model-averaged parameter estimates for variables that passed the initial screening in the double-observer experiment.

Model	Variable	Estimate	SE	85% CL	Sum of model weights
Herd size	*Herd size*	*0*.*18*	*0*.*04*	*0*.*12–0*.*24*	*1*.*00*
Front vs. rear	*Rear row*	*0*.*83*	*0*.*20*	*0*.*50–1*.*16*	*0*.*78*
Observer 2	*Observer 2*	*-0*.*41*	*0*.*23*	*-0*.*78 –-0*.*03*	*0*.*63*
Individual seat	Rear-left seat	0.63	0.28	0.17–1.08	0.22
	Front-right seat	-0.03	0.29	-0.50–0.44	0.22
	Rear-right seat	1.04	0.35	0.46–1.62	0.22
Rear-left seat	Rear-left seat	n/a			0.00

CL, confidence limits. The model-averaged estimate for rear-left seat is not shown because the variable was not present in any of the top 90% of models by weight. Variables in italics had strong support from the data.

**Fig 2:**
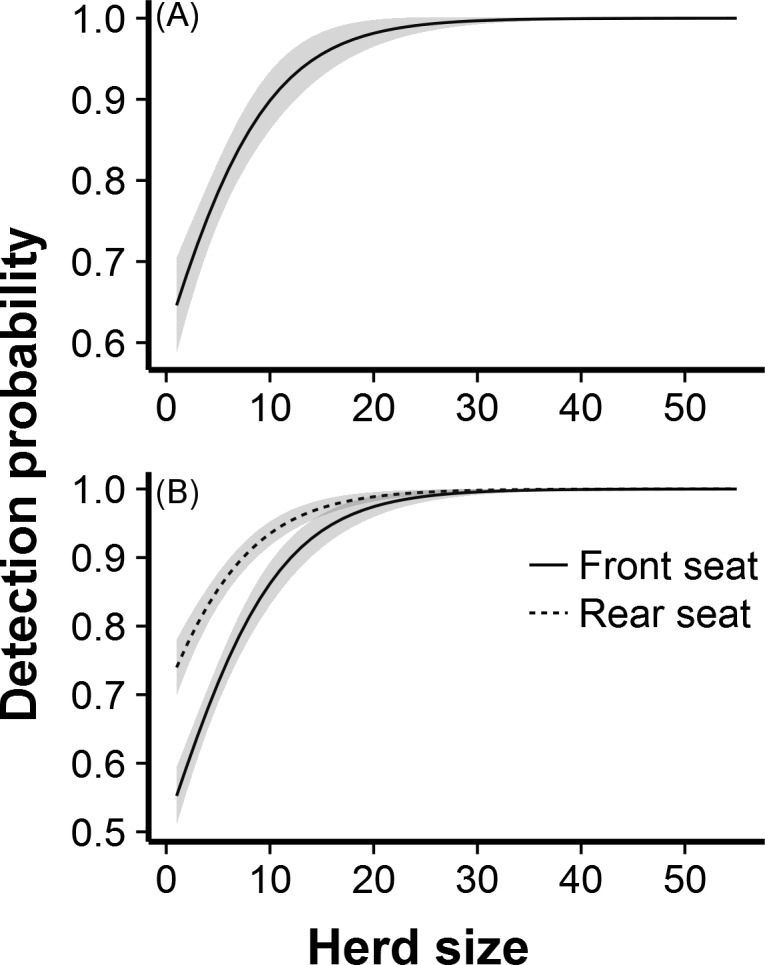
**Model-averaged effects of (A) herd size and (B) front vs. rear observer on detectability of elephants.** Shading indicates ± 1 SE. Estimates in (A) are averaged across front and rear seats. All estimates are averaged across the four observers.

**Fig 3:**
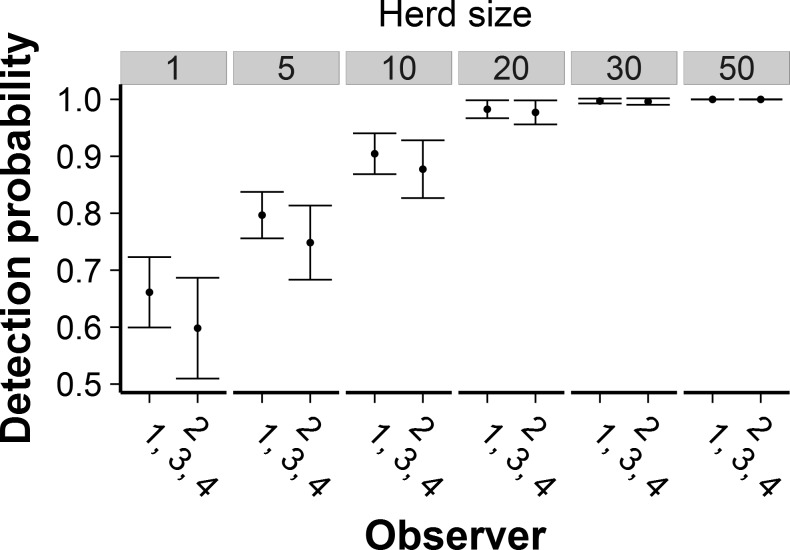
Model-averaged effect of observer on detectability of elephants for selected herd sizes. Error bars indicate ± 1 SE. Estimates are averaged over front and rear seats.

Herd size was the best-supported variable in the final models (weight = 1.0, Table 3); detection probability increased with elephant herd size (Fig 2A). Estimated detectability was 0.65 for a single elephant and increased to near 1 for herds of >25 elephants. Because herd size was so important, we plotted all other covariates in combination with it. A difference in detection probabilities between the front and rear seats had strong support, with detectability lower in the front seats (Fig 2B; Table 3). We also found strong support for observer 2 being less able to detect elephants than the other three observers (Fig 3; Table 3). For herds of >25 elephants, however, estimated detection probabilities approached 1 for all four observers. We found little support for separate detection probabilities for each seat in the plane and no support for a separate detection probability for the rear-left seat (Table 3).

**Fig 4:**
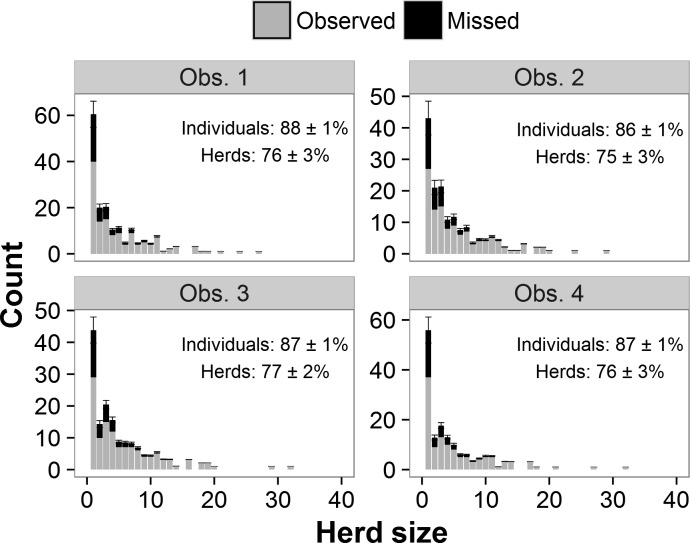
Number of elephant herds observed and estimated number missed (+ 1 SE) by herd size for each observer. Numbers in text are the estimated proportion of herds and individuals present in the survey strips that were observed on surveys (± 1 SE).

Using model-averaged parameters, we estimated that observers detected a mean of 76% ± SE of 2% of all herds and 87 ± 1% of all individuals present in the survey strips (Fig 4). Though our models showed strong support for observer 2 having a lower detection probability than the other three observers, this observer’s estimated proportions of herds and individuals detected were not significantly different from the other observers’ (herds: all |*z*| ≤ 0.80, all *P* ≥ 0.42; individuals: all |*z*| ≤ 1.06, all *P* ≥ 0.29).

**Fig 5:**
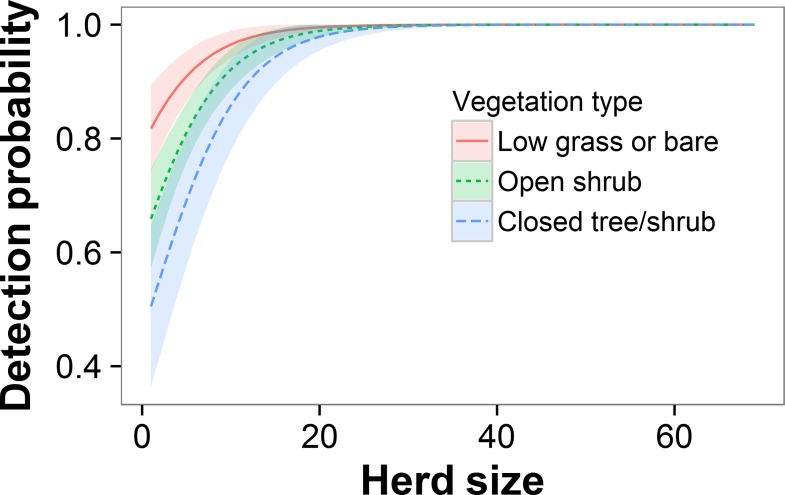
Predicted detectability of elephants by habitat type around elephant herds (± 1 SE). Values are averaged over the four observers and the front and rear seats. For readability, “tall grass” and “open tree” are not shown; detectability for those categories was very similar to “open shrub.”

For the 274 elephant herds with vegetation data, a model including five habitat categories, herd size, observer 2, and front vs. rear seat had lower AIC_c_ than a model with just herd size, observer 2, and front vs. rear seat (ΔAIC_c_ = 2.17). In the model with habitat, the parameters for closed tree/shrub, open shrub, and open tree all had 85% CI that did not include 0, indicating that detectability in those habitats is lower than in grass/bare ground (Table 4; Fig 5). The parameter estimate for tall grass was negative but had 85% CI overlapping 0 (Table 4). As with other covariates, differences in detectability between habitat categories were apparent only at low herd sizes; the model predicted that nearly all herds of >25 individuals would be detected in all habitat types (Fig 5).

**Table 4:** Parameter estimates for habitat effects on detectability in the double-observer experiment.

Parameter	Estimate	SE	85% CL
Intercept (low grass/bare ground)	0.98	0.37	0.38–1.59
Closed tree/shrub	-1.54	0.51	-2.37 –-0.70
Open shrub	-0.87	0.41	-1.54 –-0.20
Open tree	-0.94	0.45	-1.68 –-0.20
Tall grass	-0.71	0.69	-1.84–0.43

### Total counts and sample counts

**Fig 6:**
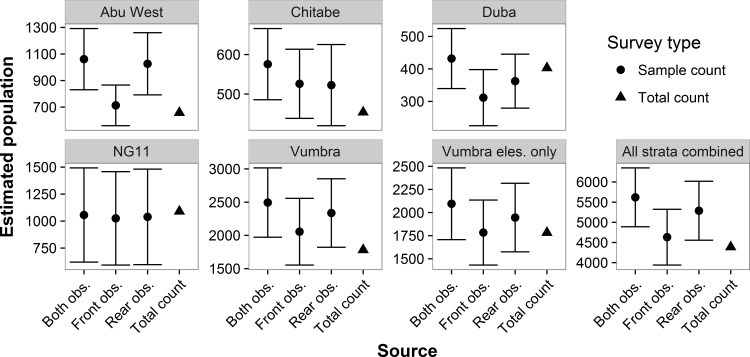
Elephant population estimates (± 1 SE) by stratum for sample and total counts. Sample-count estimates are presented for rear observers, front observers, and both rows combined on double-observer flights. The rightmost graph sums estimates from all strata but does not include the second double-observer survey of Vumbra when only elephants were counted.

We found no significant differences between total counts and sample counts for any stratum or combination of rows (front, rear, or both) on the sample counts (all |*z*| < 1.75, all *P* > 0.08; Fig 6). When we summed population estimates over all five strata, sample-count estimates were 6% greater than total-count estimates for front observers, 20% greater for rear observers, and 28% greater for both observers combined. These differences, however, were not significant (all |*z*| < 1.69, all *P* > 0.09; Fig 6).

## Discussion

Overall, we estimate that observers missed a mean of 24% of elephant herds present in the survey strips. Because, however, larger herds tended to have high detectability, the estimated proportion of individuals missed was lower at 13%. This suggests that our population estimates based on sample counts were approximately 13% below the actual values. Population estimates from sample counts were not statistically distinguishable from those based on total counts. Estimates for sample counts, however, tended to be equal to or larger than those from total counts. Because observers missed approximately 13% of elephants on sample counts, the similarity between sample and total counts suggests that total counts are biased low as well. These findings are consistent with past research indicating that observers on aerial surveys miss some large animals [11]. Even animals as large as elephants are not all detected.

Because of their large size and tendency to occur in herds, elephants should be among the most detectable species on aerial surveys. Consequently, our estimated 87% detection rate for elephants may well represent an upper limit for land mammals and suggests that other large and medium mammal species are even harder to detect. Thus, aerial surveys for mammals should assume that their results are biased low by at least 10–15% and possibly more. More research is needed to determine the amount of undercounting occurring for other species and the factors affecting their detectability.

For conservation, undercounting is important to recognize because imperfect detectability can induce spurious trends in time series [7,9]. For elephants, we can envision at least three realistic scenarios where changes in detectability may confound trend estimation. As megaherbivores, elephants change vegetation by reducing woody cover and increasing grass and herbaceous growth [39]. Hypothetically, as elephants reduce woody cover over time, their detectability would steadily increase and bias trend estimates upward. Likewise, where elephant populations have declined due to poaching, woody plant density may increase, thereby reducing detectability over time and biasing trend estimates downward. In some areas, intensive poaching of elephants has led to the formation of large aggregations, as animals appear to join together for safety [40]. This could lead to increasing detectability as populations are declining, again confounding trend estimation.

Concerns about deterministic changes in detectability are not merely hypothetical. In a recent study of antelope, a population decrease coincided with a decrease in herd size that likely reduced detectability [41]. Similarly, Hochachka & Fiedler [42] reported that the probability of resighting birds declined over time, leading to overestimation of population declines. With elephant populations declining across Africa [43,44], biased trend estimates could hinder conservation and lead to misallocation of resources. Thus, assessing elephant detectability and correcting counts for vegetation, observers, herd size, and other factors should become a standard part of survey protocols.

### Factors affecting detectability of elephants

Herd size was, by far, the most important variable affecting detectability in the double-observer study. Detectability, averaged across observers and rows, increased from 65% for a lone individual to nearly 100% for herds of >25 elephants. Herd size also influenced other covariates; effects of observer, habitat, and position in the plane were only apparent for smaller herd sizes. Large herds appear to be highly detectable regardless of other mitigating factors.

Our finding is consistent with past studies reporting that larger groups are more detectable in herd- or flock-forming animals [17,18,30,45]. Presumably, this is because larger herds offer observers more opportunities to detect at least one animal when scanning the survey strip. Because bull herds ($\bar{x}$ = 1.5 elephants in this study) tend to be smaller than breeding groups ($\bar{x}$ = 8.1 elephants), numbers of bulls are likely underestimated on many surveys, and sex ratio estimates may be biased towards females as well. Consequently, changes in sex ratios or herd sizes over time could affect the accuracy of trend estimates.

We were surprised to find a difference in detectability between front and rear observers, which has not been reported previously. In the Airvan, the front observers were immediately behind the wing struts, which may have interfered with observers’ ability to look forward for herds. Rear observers were ~0.6 m further back, so their forward view may have been less obstructed. In most aerial surveys, the observers sit in the second row of seats, immediately behind the wing struts. Our findings offer the interesting possibility that having observers sit further back in the aircraft may lead to more detections. This result, however, may be specific to the Airvan we used, as Koneff et al. [17] found that effects of seating position on detectability were aircraft-specific. Thus, effects of seating position on detectability should be tested in the specific plane models used by surveyors.

Another surprising result of the double-observer study was that the four observers missed similar proportions of elephants despite widely varying levels of experience. We found strong support for a lower detection probability for observer 2, but the estimated proportion of herds detected was just 2 percentage points lower for this observer than for the other three, and the difference was not significant (Figs 3 and 4). Observer 2 had ~150 hrs of previous survey experience. Observer 4, whose only previous experience with aerial surveys was 5 hrs of pre-study training, had detectability indistinguishable from two observers with ~1,000 hrs of previous experience. Despite these findings, we hesitate to claim that survey experience is unimportant. Studies of other species also reported significant observer effects on aerial surveys [15,17]. Observer experience may be less important for elephants than other species, but additional studies are needed to confirm this result.

The habitat immediately adjacent to elephants had a strong effect on their detections. Detectability was highest in bare ground/low grass, lowest in closed-canopy shrubs or trees, and intermediate in open trees, open shrubs, and tall grass. These findings echo a previous study reporting that detectability on aerial surveys is inversely proportional to vegetation density [13]. Despite these results, habitat is little discussed when results of aerial surveys for elephants or other mammals are reported. Perhaps this is because habitat cannot be controlled on surveys. Some ecologists suggest that raw counts, uncorrected for detectability, can be used as an index of abundance so long as detectability is roughly equal across surveys [46,47]. For factors such as observer or seat position, differences in detectability between sites or surveys may be minimal if the same observers, aircraft, and seating arrangements are used. Habitat, however, may have more insidious effects. Our data indicate that the detectability of elephants will be very different in areas with high and low woody cover. Because sample transects are usually placed systematically but use a random starting point, the actual vegetation sampled in a stratum may vary from survey to survey. In an area with heterogeneous vegetation, like the Okavango Delta, random variation in the habitats sampled can introduce statistical noise to population estimates due to differing detection probabilities. Likewise, seasonal changes in vegetation cover (e.g. wet vs. dry season) could substantially alter the detectability of elephants. Thus, comparing raw population estimates across time or space could lead to erroneous conclusions about habitat quality or population status if habitat is not considered [8].

To what extent can we generalize from our double-observer study to other elephant surveys? The ultimate goal of studies like ours is to generate correction factors to control for detectability and obtain unbiased estimates of population size. Unfortunately, survey-specific factors and the dearth of other studies examining detectability of elephants or other large mammals make applying our findings to other surveys problematic. Each type of aircraft may produce different effects of seating based on window sizes and wing strut placement [17]. Likewise, habitats will vary regionally, so that the categories used in our study may not apply universally. We did not find effects of height above ground or fatigue in our study, but other studies have, suggesting that these variables cannot be ignored based on our findings alone [15,18,30]. Our transects were relatively short ($\bar{x}$ = 16.4 km), and there was little variation in flight altitude during surveys (range = 88.7–98.1 m). Thus, our study may have had little power to detect effects of these variables. More generally, the factors that affect detectability on aerial surveys may be too variable or study-specific to apply universal corrections. Because detectability is likely to be variable across studies or even over time for the same study areas, comparisons of uncorrected population estimates may be highly misleading in some cases [8,9]. Thus, detectability analysis should be a part of each large-scale aerial survey. Ideally, each survey would produce a raw population estimate and an estimate corrected for detectability based on its own accuracy assessment.

One caveat of our analysis is that the Huggins model is useful for identifying covariates on detectability but has limitations for estimating the number of herds missed and correcting counts [48]. The model conditions on herds missed by both observers, but for missed herds, covariate values such as herd size and habitat are unknown. Thus, the model bases the estimated distribution of covariates for missed herds on that of observed herds. In reality, herds missed and herds observed should have different distributions of values for covariates affecting detectability [49]. For instance, missed elephant herds should be smaller on average than those observed (Fig 5). Bayesian data-augmentation models can be used to model unobserved covariate values [49,50]. For the top model in S2 Table, the Huggins model predicted that 21.6 ± 6.8 elephant herds were missed by both observers (unpublished results). A Bayesian data augmentation model with the same variables predicted 9.0 ± 3.7 herds missed. When one’s goal is to correct herd size estimates rather than simply to estimate detectability, data augmentation may be preferable because it explicitly models the distribution of missed herds [50,51]. We plan to explore specific methods for using double-observer counts to correct population estimates in a future publication.

### Total counts and sample counts

Before conducting this study, we hypothesized that total counts would produce higher population estimates than sample counts. We expected that improved visibility from the helicopter and its ability to hover and circle would allow observers to count elephants that were unavailable on the sample transects due to vegetation or other elephants blocking them from view. In reality, we found that total-count estimates were not significantly different from the sample counts. Sample-count estimates for front observers were 6% greater than total-count estimates, yet the Huggins model estimated that front observers missed 18% of elephants. These results suggest that total counts underestimated elephant populations roughly as much as sample counts, if not more.

Total counts generally do not have a formal sampling strip. Because, however, our total count transects were 1 km apart, observers had to search 500 m on each side of the helicopter to cover the entire stratum. Research on aerial surveys has shown that animal detections per unit area decrease rapidly as strip width increases, with detectability greatest for strips of ≤100 m [11,15]. For a 500-m wide strip, Caughley [11] found that elephant detections may be reduced by ~60%. This raises the possibility that total counts may simply require too wide a survey strip for observers to locate all herds and, therefore, may underestimate populations. In South Africa, a total count by helicopter missed a mean of 8% of elephants in a fenced population [52]. Likewise, Norton-Griffiths [19] found that total counts of African buffalo (*Syncerus caffer*) were 11% lower than the known population size. These findings support our conclusion that total counts may produce population estimates that are biased low. Total counts do not have sampling error, but our results indicate that total counts for elephants may have error due to missed herds.

One caveat with our comparison of sample and total counts is that surveys in the same stratum were separated by 4–8 days ($\bar{x}$ = 5.6 days). During this time, elephants could have moved in or out of strata, potentially affecting our population estimates. Still, we have no reason to believe that elephant movements during the intervening time were directional. The study took place in the middle of the dry season, which runs from May to October. In Botswana large-scale migrations occur at the beginning and end of the dry seasons [28,53]. Also, elephant movements in Botswana are more restricted during the dry season than at other times of year [28]. Thus, our expectation is that random movements of elephants into and out of each stratum should have been in balance and relatively infrequent. We cannot, however, rule out the possibility that small changes in the actual elephant population affected our results.

## Conclusions

Analyses and study designs that account for detectability are now *de rigueur* in a variety of disciplines within ecology and conservation [29,48]. Our results show that even species as large as elephants are subject to detectability issues. In the future, we suggest that aerial surveys for large mammals incorporate some type of detectability analysis into their methods wherever possible. Even if only a single aircraft and two observers are available, both observers can sit on the same side of the plane and conduct double-observer trials on some strata. If sufficient observers are available, one could argue that all aerial surveys should be conducted as double-observer studies. If researchers lack the expertise needed to analyze double-observer data with Huggins or data-augmentation models, the relatively simple estimator of Magnusson et al. [54] can be used. An even simpler approximation would be to use the combined observations of front and rear observers. The resulting population estimates, without any analysis of detection probabilities, will be less biased than those from a single observer. Additionally, the assumption that total counts are a complete census of a population and do not need to account for sampling error needs to be questioned. Our findings suggest that total counts may, like sample counts, be biased low, but a larger number of total and sample counts is needed to confirm this result. One useful approach would be to conduct a double-observer study within a total-count framework (i.e., unlimited strip width). Elephants are likely to be among the most detectable of all mammals, so other mammal species may have more serious detectability issues. Because population estimates from aerial surveys for elephants are biased low and vary due to study-specific factors such as observers and habitat, caution should be taken when interpreting the results of surveys and analyzing trends.

## Supporting Information

S1 Data Zip file with raw data used in double-observer analyses and in the comparison of helicopter total counts with sample counts. (ZIP)

S1 Table Results from pre-screening of variables predicting elephant detectability in double-observer aerial surveys of African elephants. See Table 1 in text for descriptions of models. Models in bold had lower AIC_c_ than a constant-only model.
(DOCX)

S2 Table Results from final set of models predicting elephant detectability in double-observer aerial surveys of elephants. (DOCX)
