# Supplementary material for: Testing the Accuracy of Aerial Surveys for Large Mammals: An Experiment with African Savanna Elephants (Loxodonta africana)
Source: PLoS One. 2016 Oct 18;11(10):e0164904. doi: 10.1371/journal.pone.0164904 (PMC5068741; doi:10.1371/journal.pone.0164904)
Supplement: S1 Table — See Table 1 in text for descriptions of models. Models in bold had lower AICc than a constant-only model. (DOCX) [file pone.0164904.s002.docx]

**S1 Table. Results from pre-screening of variables predicting elephant detectability in double-observer aerial surveys of African elephants.**

See Table 1 in text for descriptions of models. Models in bold had lower AIC_c_ than a constant-only model.

| **Category** | **Model** | **K** | **AIC_c_** | **ΔAIC_c_** |
| --- | --- | --- | --- | --- |
| Observer | **obs. 2** | **2** | **575.37** | **0.00** |
|  | (constant only) | 1 | 575.58 | 0.21 |
|  | obs. 3 | 2 | 576.21 | 0.84 |
|  | obs. 1 | 2 | 576.76 | 1.39 |
|  | obs. 4 | 2 | 577.33 | 1.96 |
|  | all 4 observers | 4 | 578.52 | 3.15 |
|  |  |  |  |  |
| Position in plane | **row** | **2** | **559.18** | **0.00** |
|  | **each position distinct** | **4** | **561.47** | **2.29** |
|  | **rear-left** | **2** | **574.09** | **14.91** |
|  | (constant only) | 1 | 575.58 | 16.40 |
|  | side | 2 | 577.59 | 18.42 |
|  |  |  |  |  |
| Fatigue | (constant only) | 1 | 575.58 | 0.00 |
|  | across days | 2 | 576.52 | 0.94 |
|  | (within day) * (within transect) | 2 | 577.52 | 1.94 |
|  | within day | 2 | 577.58 | 2.00 |
|  | within transect | 2 | 577.59 | 2.01 |
|  |  |  |  |  |
| Herd size | **herd size** | **2** | **540.10** | **0.00** |
|  | (constant only) | 1 | 575.58 | 35.48 |
|  |  |  |  |  |
| Species surveyed | (constant only) | 1 | 575.58 | 0.00 |
|  | elephants only | 2 | 577.58 | 2.00 |
|  |  |  |  |  |
| Flight parameters | (constant only) | 1 | 575.58 | 0.00 |
|  | speed | 2 | 575.88 | 0.30 |
|  | speed * height | 2 | 576.89 | 1.31 |
|  | height | 2 | 577.01 | 1.43 |
|  | direction | 4 | 578.52 | 2.94 |
|  |  |  |  |  |
| Sun position | (constant only) | 1 | 575.58 | 0.00 |
|  | relative azimuth | 2 | 577.06 | 1.48 |
|  | elevation * relative azimuth | 2 | 577.08 | 1.50 |
|  | elevation | 2 | 577.53 | 1.95 |
