# Supplementary material for: Testing the Accuracy of Aerial Surveys for Large Mammals: An Experiment with African Savanna Elephants (Loxodonta africana)
Source: PLoS One. 2016 Oct 18;11(10):e0164904. doi: 10.1371/journal.pone.0164904 (PMC5068741; doi:10.1371/journal.pone.0164904)
Supplement: S2 Table — (DOCX) [file pone.0164904.s003.docx]

**S2 Table. Results from final set of models predicting elephant detectability in double-observer aerial surveys of elephants.**

| **Model** | **K** | **AIC_c_** | **ΔAIC_c_** | **Weight** |
| --- | --- | --- | --- | --- |
| herd size + row + obs. 2 | 4 | 522.55 | 0.00 | 0.50 |
| herd size + row | 3 | 523.70 | 1.15 | 0.28 |
| herd size + position + obs. 2 | 6 | 525.27 | 2.72 | 0.13 |
| herd size + position | 5 | 526.00 | 3.45 | 0.09 |
| herd size + obs. 2 + rear-left | 4 | 537.55 | 15.00 | 0.00 |
| herd size + rear-left | 3 | 538.62 | 16.07 | 0.00 |
| herd size + obs. 2 | 3 | 539.33 | 16.78 | 0.00 |
| herd size | 2 | 540.10 | 17.55 | 0.00 |
| row + obs. 2 | 3 | 558.53 | 35.98 | 0.00 |
| row | 2 | 559.18 | 36.63 | 0.00 |
| position + obs. 2 | 5 | 561.42 | 38.87 | 0.00 |
| position | 4 | 561.47 | 38.92 | 0.00 |
| obs. 2 + rear-left seat | 3 | 573.38 | 50.83 | 0.00 |
| rear-left seat | 2 | 574.09 | 51.54 | 0.00 |
| obs. 2 | 2 | 575.37 | 52.82 | 0.00 |
| (constant only) | 1 | 575.58 | 53.03 | 0.00 |
